# Enhancing Autism Detection Through Gaze Analysis Using Eye Tracking Sensors and Data Attribution with Distillation in Deep Neural Networks

**DOI:** 10.3390/s24237792

**Published:** 2024-12-05

**Authors:** Federica Colonnese, Francesco Di Luzio, Antonello Rosato, Massimo Panella

**Affiliations:** Department of Information Engineering, Electronics and Telecommunications (DIET), University of Rome “La Sapienza”, Via Eudossiana 18, 00184 Rome, Italy; federica.colonnese@uniroma1.it (F.C.); francesco.diluzio@uniroma1.it (F.D.L.); antonello.rosato@uniroma1.it (A.R.)

**Keywords:** autism spectrum disorder, gaze analysis, eye tracking sensors, deep neural networks, explainability, TracIn method

## Abstract

Autism Spectrum Disorder (ASD) is a neurodevelopmental condition characterized by differences in social communication and repetitive behaviors, often associated with atypical visual attention patterns. In this paper, the Gaze-Based Autism Classifier (GBAC) is proposed, which is a Deep Neural Network model that leverages both data distillation and data attribution techniques to enhance ASD classification accuracy and explainability. Using data sampled by eye tracking sensors, the model identifies unique gaze behaviors linked to ASD and applies an explainability technique called TracIn for data attribution by computing self-influence scores to filter out noisy or anomalous training samples. This refinement process significantly improves both accuracy and computational efficiency, achieving a test accuracy of 94.35% while using only 77% of the dataset, showing that the proposed GBAC outperforms the same model trained on the full dataset and random sample reductions, as well as the benchmarks. Additionally, the data attribution analysis provides insights into the most influential training examples, offering a deeper understanding of how gaze patterns correlate with ASD-specific characteristics. These results underscore the potential of integrating explainable artificial intelligence into neurodevelopmental disorder diagnostics, advancing clinical research by providing deeper insights into the visual attention patterns associated with ASD.

## 1. Introduction

In recent years, Neural Networks (NNs) have gained significant importance in the classification and diagnosis of medical conditions: their ability to process large amounts of complex, high-dimensional data, such as images, time series, or sensor recordings, on a scale unreachable by traditional methods, makes them particularly well-suited for the medical field where diagnoses are often complex and multi-dimensional [1]. This is particularly relevant for neurodevelopmental disorders like Autism Spectrum Disorder (ASD) and Attention Deficit Hyperactivity Disorder (ADHD), where current official diagnostic protocols, based on the Diagnostic and Statistical Manual of Mental Disorders (DSM-5) [2], rely heavily on subjective tools such as behavioral observations, standardized questionnaires, and interviews with parents and teachers.

Specifically, the diagnostic process for ASD focuses on identifying deficits in social communication and interaction, along with restricted and repetitive patterns of behavior [2]. However, these approaches come with significant drawbacks: their accuracy often depends on the clinician’s expertise and the individual’s responses, leading to variability in the diagnostic outcomes. Moreover, these tools frequently fail to incorporate the latest advancements in clinical research, which highlights the need for more objective, data-driven methods. This reliance on subjective assessments reduces diagnostic precision and consistency, creating challenges for early detection and timely interventions.

Incorporating objective data sources that align with emerging clinical evidence, such as eye tracking metrics [3], gait analysis [4], and other behavioral or physiological measurements [5,6], could provide more reliable and data-driven diagnoses. By leveraging the power of Deep Learning (DL), these models could be integrated into the diagnostic process and enhance current practices by integrating objective biomarkers with clinical expertise: rather than replacing official diagnostic techniques, they are designed to support the overall diagnostic process, for example, serving as screening tools in schools or as decision support systems for clinical staff. The ultimate goal is to enhance both precision and efficiency, leading to faster and more accurate diagnoses. By reducing diagnostic times and clinician workload, this approach aims to make the diagnostic process more accessible, streamlined, and reliable.

As mentioned earlier, among the physiological and behavioral data sources currently being explored, eye tracking has shown particular promise in supporting ASD diagnosis: eye tracking technology allows for the precise measurement of gaze patterns, providing crucial insights into visual attention behaviors [7]. Research has consistently shown that individuals with ASD often exhibit atypical gaze patterns, such as a reduced focus on socially relevant stimuli, including faces and eyes, in favor of peripheral or object-focused attention [8], which could be used as a potential biomarker for early diagnosis. Integrating eye tracking data such as scanpaths and fixation maps into DL models can refine the diagnostic process by introducing objective methods that complement traditional clinical assessments. This approach helps detect early indicators of ASD and enhances both the accuracy and efficiency of the diagnostic workflow.

Although NNs offer significant advantages in analyzing large datasets, the computational cost and time required can become overwhelming when processing extremely large volumes of data. To address these challenges, data attribution methods have emerged as valuable tools in Machine Learning (ML): they provide insights into the inner workings of complex models, shedding light on the factors that drive predictions at the individual sample level, by quantifying the influence of specific data points, enhancing the interpretability and trustworthiness of otherwise “black-box” models. Several data attribution methods have been proposed, such as Trak [9], SimFluence [10], and Datamodels [11]. In this paper, we use TracIn [12], a method that traces the model’s behavior back to its training data by leveraging loss gradients to calculate influence scores. These scores help assess relationships between training and test samples and can also uncover anomalies or mislabeled data points within the training set, making TracIn especially useful for improving model performance on large-scale datasets. Combining in this way the strengths of DL and data attribution methods enables the creation of more efficient, interpretable, and reliable diagnostic models for conditions like ASD, holding promise for both clinical and computational fields.

In this paper, three different NNs combined were employed to classify ASD using scanpaths, fixation maps, and images data derived from eye tracking experiments [13]. The primary aim was to accurately classify individuals with ASD from neurotypical controls by analyzing their unique visual attention patterns and, to further improve both model performance and interpretability, TracIn [12] was used. Two key functionalities of TracIn were employed: firstly, self-influence was evaluated to identify outliers in the dataset, which were subsequently removed, improving both model accuracy and training times; secondly, influence scores were applied to assess which training images, and their associated scanpaths, had the most significant impact on the classification of ASD. This process helped to identify the most representative visual patterns for the ASD class, offering a deeper understanding of the model’s behavior and its ability to distinguish ASD-related gaze behaviors from those of neurotypical individuals, which is consistent with medical evidence.

Our experimental results demonstrate a significant improvement in classification performance compared to previous methods, achieving a test accuracy of 94.35% and an F1-score of 93.15% on the “Saliency4ASD” dataset [13]. By filtering out noisy or anomalous data points using self-influence scores, we not only enhanced model accuracy but also reduced computational costs. Additionally, our analysis highlights that images featuring people or groups of people are the most influential in distinguishing ASD-related gaze patterns from neurotypical behaviors. This finding aligns with existing clinical research, further supporting the model’s ability to identify key visual cues relevant to ASD diagnosis.

The main contributions of this paper are as follows:

1. Efficient Classification with Limited Fixation Points. An NN architecture is presented that effectively handles scanpaths with a limited number of fixation points, which are often sparse in eye tracking data. By applying data augmentation techniques to these fixation points (as detailed in Section 3), a more diverse set of scanpaths is generated. This augmentation enhances the model’s ability to generalize across various scanpath patterns, leading to improved accuracy and robustness in ASD classification.
2. Incorporation of TracIn for Dataset Distillation. TracIn [12], a data attribution method, is incorporated into the classification process for ASD detection using NNs. This enables the identification and removal of non-contributory or anomalous samples, enhancing the quality of the training dataset: as a result, classification efficiency and accuracy are improved by focusing on the most relevant and influential data points.
3. Optimized Performance and Reduced Computational Costs. By refining the dataset on the most significant training examples, this approach boosts overall classification performance while reducing computational costs. This targeted training not only leads to more accurate predictions but also optimizes resource usage, making the process more efficient for large-scale classification tasks.
4. Comprehensive Explainability and Data Insights. The proposed method offers a detailed explainability analysis, providing insights into the influence of specific images from the training data on the ASD class. This transparency aids in understanding how individual samples contribute to the model’s predictions. Additionally, the approach captures critical patterns and relationships within the data, facilitating better management of large-scale datasets and helping to inform decisions in both the training and deployment stages.

These contributions demonstrate the effectiveness of integrating data attribution techniques, such as TracIn, in improving the accuracy, efficiency, and interpretability of neural network-based classification models, particularly for ASD diagnosis.

The rest of the paper is structured as follows. Section 2 reviews related work on the application of NNs in medical diagnostics, with a focus on ASD detection, and discusses the importance of explainability in ML and DL models for clinical applications. Section 3 provides an overview of the dataset, and details the preprocessing techniques applied, while Section 4 details the proposed methodology, including the NN architecture and the use of TracIn for outlier detection and interpretation. Lastly, Section 5 presents the experimental setup and results, discussing the impact of outlier removal on model performance, and Section 6 concludes the paper with key insights and directions for future research.

## 2. Related Works

The use of NNs in medical diagnostics has gained considerable attention in recent years, driven by their ability to process large, complex datasets and identify patterns that are often elusive to traditional diagnostic methods [14]. From image recognition in radiology [15] to the outcome prediction of patients with tumors [16], DL models have shown remarkable potential in identifying patterns and correlations that may not be apparent through traditional methods. Studies have demonstrated their effectiveness across various domains, including oncology [17], cardiology [18], and detection of depression [19,20], where they aid in early diagnosis and decision support systems. This revolution in healthcare data processing has transformed how medical professionals can interpret data, offering more accurate and faster diagnostic tools.

### 2.1. Neural Networks and ASD Classification

In the context of neurodevelopmental disorders, such as ASD and ADHD, NNs are increasingly being employed to analyze complex behavioral data, leveraging datasets that align with clinical evidence and medical research on how specific tools could aid in their diagnosis. For example, brain imaging techniques such as Functional Magnetic Resonance Imaging (fMRI) have been used in combination with DL models [21,22] to accurately identify individuals with ADHD, as studies have demonstrated notable differences in their brain activity compared to neurotypical individuals [23,24]. Additionally, Electroencephalography (EEG) has been employed in the detection of ASD using NNs [25,26], as it has been shown [27] that EEG signals can effectively be used as a biomarker to differentiate individuals with ASD from neurotypical individuals.

In addition to medical tools like fMRI and EEG, other physiological and behavioral features have been explored for classifying ASD. Gait analysis, for example, has been used in DL studies to classify autism based on walking patterns, as the DSM-5 [2] identifies these as associated features of ASD. Research shows that individuals with ASD often exhibit distinct gait characteristics, which can serve as valuable biomarkers for diagnosis [28,29,30]. Similarly, eye gaze patterns have proven to be reliable indicators of autism: eye tracking technology offers a non-invasive and objective way to study visual attention, and research has consistently demonstrated that children with ASD tend to focus less on faces during social interactions, instead directing their attention to less socially informative areas of a scene [31]. For instance, a novel framework for ASD screening is introduced in [32] for integrating information from multiple behavioral modalities, such as photo-taking and image-viewing tasks, to enhance classification performance. They created a reliable model that classifies people with ASD using a photo-taking task where subjects freely explore their environment in a more ecological setting. By leveraging temporal information in eye movements, the framework achieves an accuracy of 99%, outperforming other state-of-the-art methods. These insights have led to the integration of eye tracking data into DL models, where gaze patterns—represented as scanpaths and fixation maps—are used to train classifiers for ASD detection. In the following section, we will use a state-of-the-art model as a benchmark to compare our results with those of other models implemented on the same dataset, as summarized in Section 5.1.

In [33], the authors propose “RM3ASD”, a framework that extracts features from both raw images, used as stimuli in eye tracking experiments, and scanpaths containing gaze and temporal data. To optimize their model, they experimented with 23 different classifiers, with the TreeBagger classifier achieving the highest accuracy of approximately 68.5%.

Another method is presented in [34], where the model “SP-ASDNet” combines a CNN for feature extraction and an LSTM network to analyze the temporal information of fixation points, aiming to classify observers as either typically developing (TD) or having ASD. SP-ASDNet achieved a validation accuracy of 74.22%, but its test accuracy dropped to 57.90%, indicating potential overfitting.

Two DL methods for detecting autism-related gaze patterns during free viewing are introduced in [35]. The first, “STAR-FC”, uses generative synthetic saccade models with auxiliary data, while the second method transforms real scanpaths and image stimuli into a unified representation. Their models achieved validation accuracies of up to 67.23% and test accuracies of 61.39% for ASD prediction.

Finally, in [32], the authors introduced a novel ASD screening approach using two DNNs. One network combined ResNet-50 and LSTM for processing photo sequences, while the other analyzed eye movements during image viewing. Their multi-modal approach improved accuracy from 89% to 93% on the “Saliency4ASD” dataset.

### 2.2. Data Attribution in NNs

The increasing use of NNs in healthcare has raised concerns about their black-box nature, where the internal decision-making processes are often opaque. This lack of transparency has made explainability a crucial requirement for AI models, particularly in healthcare, where understanding and trusting predictions are vital for both clinicians and patients. Explainability methods, specifically Training Data Attribution (TDA), have emerged to address this issue by evaluating the influence of individual training data points on a model’s predictions, providing insights into how specific data contribute to the model’s output.

TDA is especially useful in sensitive fields like neurodevelopmental disorders, where explainability and trust in AI are critical for successful clinical integration. These methods are generally categorized into two approaches: retraining-based and gradient-based methods [36]. The first ones, such as downsampling [37], assess the impact of different training subsets on model performance, offering comprehensive insights but at high computational costs, making them impractical for large datasets or complex models. In contrast, gradient-based methods are more efficient, leveraging gradients from the loss function to compute influence scores. These methods, like TracIn [12], track changes in loss on test points throughout training, offering scalable solutions with lower computational overhead.

In neurodevelopmental disorder diagnosis, TDA methods enhance model explainability by identifying which training examples most strongly influence predictions, offering transparency in how disorders such as ASD are classified. Aligning these results with clinical research not only validates the models but also demonstrates that AI can complement ongoing clinical progress. Recent work has successfully integrated TDA with ASD detection. For instance, DL with explainability methods using eye tracking data are combined in [38] to bridge the gap between clinical research and model predictions. Another study in [39] employs a hybrid DL approach to identify key ASD-related features for improving early diagnosis, while SHAP explanations have demonstrated how analyzing interaction features in home videos can support early ASD identification, aiding early interventions [40].

## 3. Dataset Overview and Preparation

Eye tracking provides a non-invasive, quantifiable tool for studying attentional differences, and it is widely used in clinical research [41]. In this study, eye gaze tracking was chosen as the primary method due to its proven effectiveness in identifying differences in visual attention patterns between ASD and TD children, as explained in Section 2.1.

**Figure 1:**
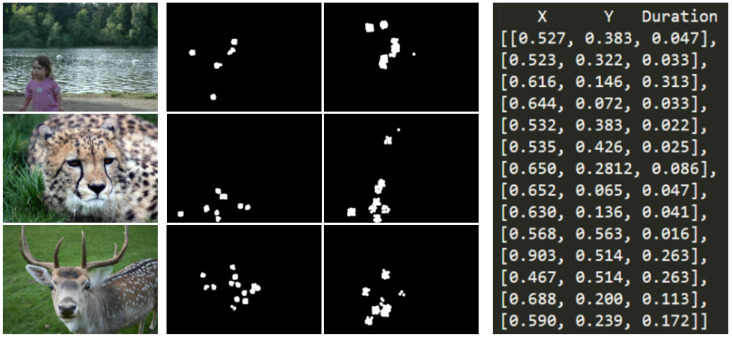
Samples from the Saliency4ASD dataset used for training the neural network model, which include images, fixation maps, and scanpaths.

The dataset used in this paper, described in [42] and published in 2018, contains eye movement data recorded using the “Tobii T120 Eye Tracker^®^” sensor, from 14 children with ASD and 14 neurotypical controls (TD) with an age between 5 and 12. It includes 600 scanpaths, fixation maps, and heatmaps across 300 visual stimuli, categorized into objects, people, landscapes, and more, as shown in Figure 1, and participants looked at each of them for 3 s.

The original dataset provided two sets of data for each image: one for the ASD group and one for the TD group. Each set included scanpaths, fixation maps, and heatmaps, aggregating all fixation points for the respective class. However, this setup is not ideal for practical applications, since the goal is to detect ASD at the individual level, rather than analyzing a collective group of children within the same diagnostic category. Additionally, aggregating scanpaths from multiple participants masks individual differences and patterns, which are critical for training a model to accurately identify ASD in a single child. Therefore, a number of preprocessing steps were applied to make the dataset compliant with the scope of this research paper.

The first preprocessing step involved decomposing the aggregated scanpath files into individual scanpaths, denoted as $s_{i,j}$, where *i* represents the participant and *j* the specific image viewed, producing $N_{j}\times j$ scanpaths across both classes (ASD and TD), where $N_{j}$ is the number of participants that saw that specific image. For each scanpath $s_{i,j}$, the corresponding image *j* was replicated, ensuring that each scanpath had its paired image. This decomposition was straightforward because the scanpath files included indexes indicating the number of fixation points for each participant. A new scanpath started at each index 0, marking the beginning of a new participant’s data. To ensure the rationality and validity of this approach, it is important to note that the decomposition result is unique. The provided indexes and fixation counts for each participant allowed for an unambiguous mapping of aggregated scanpaths to individual participants. A data augmentation process was applied to address the shortness of single scanpaths, which often contained very few fixation points (sometimes as few as two). Each scanpath is defined by three columns $\left(x_{i},y_{i},d_{i}\right)$, where $x_{i}$ and $y_{i}$ are the spatial coordinates of the fixation, and $d_{i}$ is the duration. Durations were initially in milliseconds and were rescaled between 1 and 100. Then, the points were then replicated according to their scaled duration, and a random shift Δx,Δy∈[−10,10] was applied to each fixation point to capture a broader area. Lastly, a similar preprocessing approach was applied to the fixation maps, originally provided in aggregated form. They were aligned with the individualized scanpaths: each fixation map was recreated with a radius of 10 pixels, expanding the region of interest to the hole beyond a single gaze point. This augmentation process serves several purposes. Firstly, this transformation improved the density of the scanpaths and aimed to make the gaze patterns more informative, ensuring that even short scanpaths provide richer information for the model during training. Secondly, the random spatial shifts introduce natural variability, reflecting slight changes in gaze patterns that are typical in real-world scenarios. Importantly, this augmentation preserves the original structure and temporal dynamics of the gaze behavior, as the replication is directly proportional to the fixation duration.

## 4. Methodology

**Figure 2:**
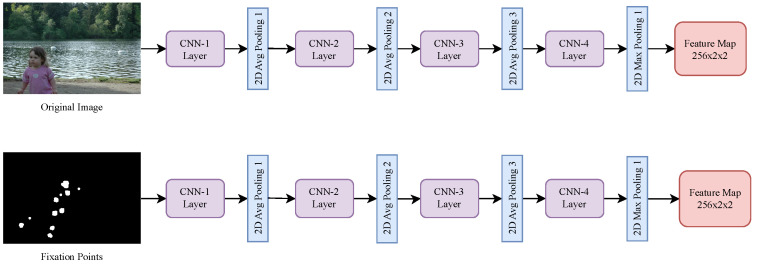
Architecture of the first two sub-networks (CNN_image_FM, CNN_fixmaps_FM), which processes the input images and fixation maps to extract spatial features for ASD classification.

**Figure 3:**
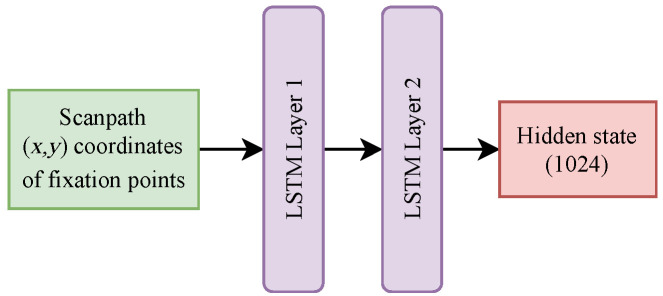
Architecture of the LSTM_scanpath_HS network used for scanpath sequences, capturing the temporal dependencies between fixation points.

**Figure 4:**
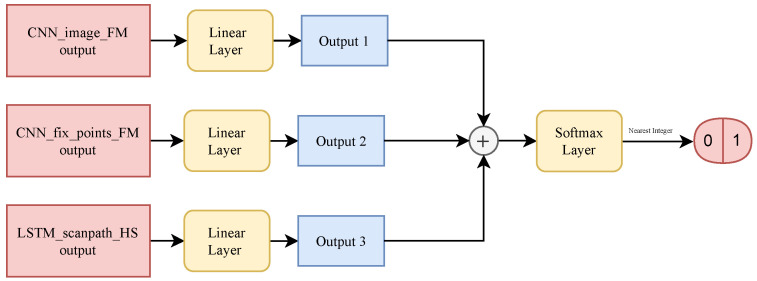
Overview of the complete GBAC model, illustrating the concatenation of the outputs from the image, fixation point, and scanpath sub-networks for the final classification.

The proposed method, which will be referred to in the following as “Gaze-Based Autism Classifier” (GBAC), is illustrated in Figure 2, Figure 3 and Figure 4, and its hyperparameters are summarized in Table 1. GBAC integrates TracIn into the diagnosis of ASD through an NN that takes eye gaze data as input. GBAC comprises three sub-networks: a Convolutional Neural Network (CNN) for processing the original image, a CNN for fixation points, and an LSTM for scanpath coordinates. Once trained, TracIn is applied to evaluate self-influence, helping to identify outliers that negatively affect model performance; these outliers are then removed from the dataset, and the model is retrained on the refined subset. Finally, TracIn is used to identify proponents and opponents for the test set images, highlighting the most influential data points for classifying ASD, which helps in understanding key differences between ASD and neurotypical cases.

### 4.1. Neural Network Architecture

The NN architecture developed for the classification of ASD based on eye tracking data integrates three primary sub-networks: one for processing images, one for fixation points, and one for scanpaths, effectively capturing both spatial and temporal aspects of the data. The first sub-network, called “CNN_images_FM”, is a CNN that processes the images shown to participants: this CNN extracts spatial features from the images through multiple convolutional layers, each applying a filter to the input and passing the result through a non-linear Rectified Linear Unit (ReLU) activation function. The output feature maps capture the essential visual characteristics of the images, denoted as ${\mathrm{CNN}}_{\mathrm{img}}\left(I_{j}\right)$, where $I_{j}$ is the input image for a particular participant. The second sub-network is another CNN called “CNN_fixmaps_FM”, tailored for the fixation points captured during eye tracking: each fixation point is characterized by its spatial coordinates $\left(x_{i},y_{i}\right)$. Similar to the image CNN, this sub-network applies a series of convolutional layers to extract meaningful features from the fixation data, producing an output denoted as ${\mathrm{CNN}}_{\mathrm{fix}}\left(x_{i},y_{i}\right)$. This sub-network captures the spatial relevance of fixation points across the images. The architectures of the first two sub-networks are shown in Figure 2. The “CNN_images_FM” and “CNN_fixmaps_FM” sub-networks were designed with similar architectures to efficiently extract spatial features from their respective inputs: raw images and fixation maps. Although the inputs differ, the underlying task of spatial feature extraction is consistent, making the shared structure suitable for both. Specifically, the “CNN_fixmaps_FM” network extracts spatial relevance from fixation patterns, such as density and distribution, while the “CNN_images_FM” network processes detailed visual features within the raw images. This decision also aligns with our aim to maintain simplicity and practicality in the architecture. For real-world applications, efficient and robust designs are crucial.

In order to handle the temporal nature of the scanpath data, the third sub-network, called “LSTM_scanpath_HS”, utilizes a Long Short-Term Memory (LSTM) network, as shown in Figure 3. Scanpaths, defined as sequences of fixation points $\left(x_{1},y_{1}\right),\cdots ,\left(x_{k},y_{k}\right)$, are fed into the LSTM, which processes these sequential points over time, maintaining a hidden state that captures the temporal dependencies. At each time step, the LSTM updates its hidden state, and the final hidden state $h_{T}$ encapsulates the entire scanpath sequence, represented as ${\mathrm{LSTM}}_{\mathrm{scnpts}}\left(s_{i,j}\right)$.

After the outputs of these three sub-networks are generated—${\mathrm{CNN}}_{\mathrm{img}}\left(I_{j}\right)$, ${\mathrm{CNN}}_{\mathrm{fix}}\left(x_{i},y_{i}\right)$, and ${\mathrm{LSTM}}_{\mathrm{scnpts}}\left(s_{i,j}\right)$—each output is passed through its linear layer, which reduces its dimensionality to 1. Specifically, the transformations are as follows:

(1) $$\begin{array}{cc} \; & O_{\mathrm{img}}=\mathrm{Linear}\left[{\mathrm{CNN}}_{\mathrm{img}}\left(I_{j}\right)\right]\;, \end{array}$$

(2) $$\begin{array}{cc} \; & O_{\mathrm{fix}}=\mathrm{Linear}\left[{\mathrm{CNN}}_{\mathrm{fix}}\left(x_{i},y_{i}\right)\right]\;, \end{array}$$

(3) $$\begin{array}{cc} \; & O_{\mathrm{scnpts}}=\mathrm{Linear}\left[{\mathrm{LSTM}}_{\mathrm{scnpts}}\left(s_{i,j}\right)\right]\;. \end{array}$$

These three outputs ($O_{\mathrm{img}}$, $O_{\mathrm{fix}}$, and $O_{\mathrm{scnpts}}$) are then concatenated along the dimension *x*. The concatenated vector is passed through a final linear layer that performs the classification, producing the output probability $\hat{y}$:

(4) $$\hat{y}=\mathrm{Softmax}\left\{\mathrm{Linear}\left[\left(O_{\mathrm{img}},O_{\mathrm{fix}},O_{\mathrm{scnpts}}\right)\right]\right\}\;.$$

The GBAC architecture, illustrated in Figure 4, was intentionally divided into three different sub-networks, each one designed to capture a specific type of information relevant to the classification task; in this way, this separation allows for a more targeted extraction of features that are crucial for distinguishing between ASD and TD classes. The first sub-network (presented in Figure 2) processes the original images as input, focusing on the primary visual elements within the scene, ensuring that the model captures the fundamental characteristics of the image that might influence gaze behavior. The second sub-network (presented in Figure 2) takes fixation maps as input: by isolating fixation patterns from the original image, this sub-network aims to identify visual patterns that differentiate ASD and TD individuals, independent of the raw image content. The third sub-network (presented in Figure 3) processes scanpaths, introducing a temporal dimension to the analysis, and providing insights into how visual information is processed over time in ASD and TD individuals.

Each sub-network is followed by a dedicated fully connected layer (presented in Figure 4) to independently assess the contribution of its respective input modality; the outputs of these fully connected layers are then concatenated and passed through a final fully connected layer to combine the insights from all three sub-networks. This design ensures that each sub-network output is individually processed before combining them for the final classification, allowing the model to separately handle spatial and temporal data before integrating them into the final decision-making layer. Such an approach optimizes the model’s ability to capture both the spatial and temporal aspects of gaze behavior, resulting in more accurate ASD detection.

### 4.2. TracIn Formulation

TracIn is a method designed to estimate the influence of individual training samples on the model’s predictions by approximating the impact that each sample has on the final trained model; it achieves that by leveraging gradient-based techniques to compute influence scores, offering a scalable and computationally efficient way to assess the significance of training data throughout the learning process.

To reduce the computational cost of exact influence computation, TracIn uses a first-order gradient approximation by replaying the training process across multiple checkpoints, which store model states at different points during training. For each training sample, the gradient of the loss function concerning the model parameters is calculated at these checkpoints. The influence score is then derived by accumulating the inner product of these gradients across the checkpoints.

Given the loss function *l* of the model, a set of *k* checkpoints, learning rates $\eta_{i}$, model parameters $w^{\left(i\right)}$ at checkpoint *i*, and a training sample *x*, the self-influence score measuring how much a sample influences its own prediction is computed as follows:

(5) $$\mathrm{Self}-\mathrm{Influence}\left(x\right)=\sum_{i=1}^{k}\eta_{i}\nabla l\left(w^{\left(i\right)},x\right)\cdot \nabla l\left(w^{\left(i\right)},x\right)\;.$$

The self-influence score reflects the extent to which a specific training sample impacts the model’s weights during training: samples with higher self-influence values are often outliers, mislabeled, or exhibit conflicting behavior, negatively affecting the model’s generalization capability and are typically removed to improve overall performance.

The basic model trained on the entire dataset will be referred to as “GBAC-FT”. However, in our approach, after computing the self-influence score for each training sample, we remove the ones with the highest scores as they are likely to hinder the model’s ability to generalize. This filtering process results in a more representative and concise training set, leading to both improved accuracy and enhanced computational efficiency. The model obtained on a reduced dataset is referred to as “GBAC-SIST”. For comparison purposes, we also retrain the model using the same number of samples on a subset chosen randomly, denoted as “GBAC-RST”. It is important to note that the self-influence filtering is applied exclusively to the training set, while the test set remains unchanged across all experiments. This ensures a fair comparison by evaluating each model on the same test data, thereby maintaining the consistency and validity of the reported performance metrics.

In addition to self-influence, TracIn is also employed to calculate the influence of training samples on test samples, helping to identify which training examples are most relevant for correct classification. Specifically, the influence of a training sample *x* on a test sample $x_{\mathrm{test}}$ is determined by computing the inner product between the loss gradients of the training and test samples:

(6) $$\mathrm{Influence}\left(x,x_{\mathrm{test}}\right)=\sum_{i=1}^{k}\eta_{i}\nabla l\left(w^{\left(i\right)},x\right)\cdot \nabla l\left(w^{\left(i\right)},x_{\mathrm{test}}\right)\;.$$

High influence scores indicate that a training sample strongly supports the correct classification of the test sample (proponents), while negative influence scores may point to conflicting or misleading training data (opponents). By analyzing the most influential training samples for each class, we can identify those that best represent the key characteristics of both ASD and TD groups. This process further refines the model, improving its performance and providing deeper insights into the relationships between training and test data.

## 5. Experiments

Several experiments were conducted to evaluate the efficiency of the proposed methodology: the experimental setup, along with numerical and visual results, are presented in the following sections. All the results reflect the average of *r* independent runs, with r=5, each utilizing a different random seed to ensure robustness. The three models composing GBAC, presented in Section 4.1, were selected after conducting a comprehensive grid search to determine the optimal architecture and hyperparameters for each sub-network. All the experiments were performed using Python and Pytorch^®^ backend on a machine equipped with an AMD Ryzen^™^ 7 7700 8-core CPU at 3.80 GHz, 32 GB of RAM, and an NVIDIA^®^ GeForce^™^ RTX 4080 SUPER GPU at 16376 MB of GDDR6X RAM.

### 5.1. Experimental Results

**Figure 5:**
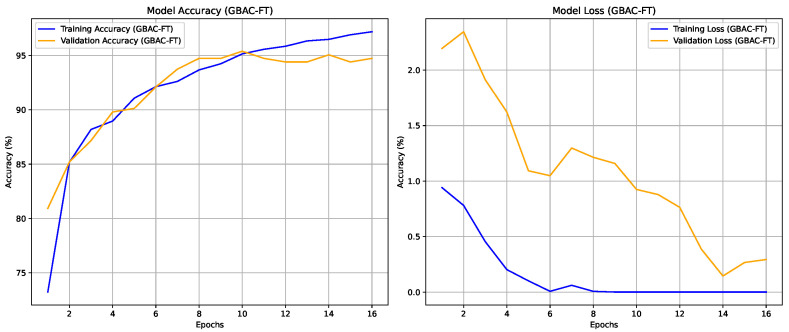
Accuracy and loss trends for training and validation sets across 16 epochs for model GBAC-FT.

The experiments were conducted to validate the effectiveness of the proposed GBAC model for classifying ASD based on eye tracking data. Initially, the performance of the GBAC-FT (summarized in Figure 5) model trained on the entire dataset was compared with models trained on both random and self-influence-reduced subsets of the training data. A key aspect of our method was the use of TracIn to compute self-influence scores, allowing us to filter out high-influence training samples, which were typically noisy or outliers, as explained in Section 4.2. We applied three different thresholds for dataset reduction, removing samples with self-influence scores above each respective threshold. The suffix “1”, “2”, or “3” is appended to both the GBAC-SIST and GBAC-RST models, denoting a dataset usage of 77%, 67%, and 50%, respectively.

To provide a comprehensive understanding of the evaluation metrics used in the experiments, the metrics used are define as follows.

Test Accuracy measures the proportion of correctly classified samples in the test dataset, and it is defined as follows:

(7) $$\mathrm{Test}\;\mathrm{Accuracy}=\frac{\mathrm{Number}\;\mathrm{of}\;\mathrm{Correct}\;\mathrm{Predictions}}{\mathrm{Total}\;\mathrm{Number}\;\mathrm{of}\;\mathrm{Test}\;\mathrm{Samples}}$$

Precision, F1-Score, and Recall are used to evaluate additional information on the test results and are defined as follows:

(8) $$\mathrm{Precision}=\frac{\mathrm{True}\;\mathrm{Positives}\;\left(\mathrm{TP}\right)}{\mathrm{True}\;\mathrm{Positives}\;\left(\mathrm{TP}\right)+\mathrm{False}\;\mathrm{Positives}\;\left(\mathrm{FP}\right)}$$

(9) $$\mathrm{Recall}=\frac{\mathrm{True}\;\mathrm{Positives}\;\left(\mathrm{TP}\right)}{\mathrm{True}\;\mathrm{Positives}\;\left(\mathrm{TP}\right)+\mathrm{False}\;\mathrm{Negatives}\;\left(\mathrm{FN}\right)}$$

(10) $$F1-\mathrm{Score}=2\cdot \frac{\mathrm{Precision}\cdot \mathrm{Recall}}{\mathrm{Precision}+\mathrm{Recall}}$$

**Figure 6:**
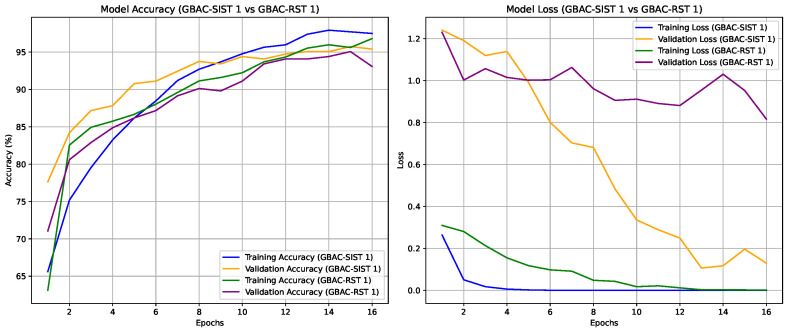
Accuracy and loss trends for training and validation sets across 16 epochs for models GBAC-SIST 1 and GBAC-RST 1.

The results, summarized in Table 2, indicate that the Self-Influence Selected Training GBAC-SIST models consistently outperformed the GBAC-RST versions across all configurations. The accuracy of GBAC-SIST improved from 93.18% to 94.35%, and the F1-score increased from 0.9094 to 0.9315. Notably, the GBAC-SIST1 variant achieved the highest test accuracy of 94.35% and an F1-score of 0.9315 while using only 77% of the dataset. In contrast, GBAC-RST1, which used the same amount of training data selected randomly, showed lower performance, emphasizing the advantage of self-influence-based selection, as can be seen in Figure 6.

**Figure 7:**
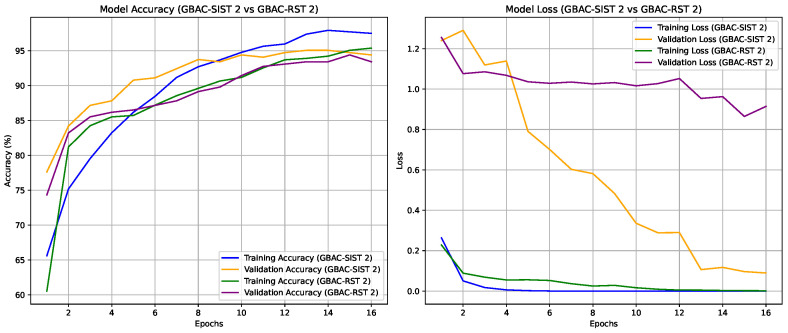
Accuracy and loss trends for training and validation sets across 16 epochs for models GBAC-SIST 2 and GBAC-RST 2.

**Figure 8:**
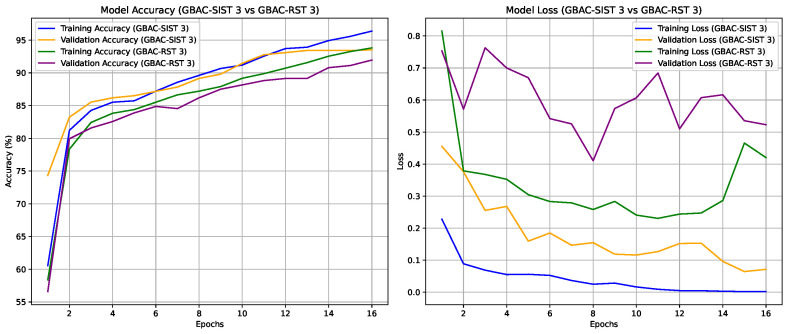
Accuracy and loss trends for training and validation sets across 16 epochs for models GBAC-SIST 3 and GBAC-RST 3.

Interestingly, even smaller training subsets selected based on self-influence performed better than models trained on the full dataset, as can be seen in Figure 7 and Figure 8. For instance, GBAC-SIST2, which used only 67% of the training data, achieved an accuracy of 94.13%, surpassing GBAC-FT trained on the whole dataset. This highlights the benefit of removing noisy or conflicting samples identified by TracIn’s self-influence scores, as it not only boosts performance but also reduces the training set’s size, improving training efficiency and model robustness. This result underscores the advantage of self-influence filtering, which enhances generalization by removing noisy or conflicting data points that could interfere with model learning. This kind of metrics are important to use since the understanding of eye movement dynamics in ASD subjects is still evolving in clinical research, and so it is not feasible to visually determine whether the samples removed via the self-influence calculation are indeed noise or outliers. The main purpose of evaluating self-influence scores is to systematically identify samples that negatively impact model generalization, without relying on subjective criteria. The effectiveness of this approach is also supported, as shown in this section, by the improved model performance observed when samples are removed based on self-influence scores, compared to random removal.

Results are also benchmarked against existing models in the literature, such as RM3ASD [33], SP-ASDNet [34], and STAR-FC [35], with performance metrics presented in Table 3 against the GBAC-SIST1 version that outperformed the other proposed variants. All experiments were performed using the Saliency4ASD dataset [13], consisting of 2033 samples evenly divided between the two classes, by the preprocessing steps detailed in Section 3. The proposed GBAC model is able to obtain the best performance over all of the metrics with a consistent gap with respect to the other considered approaches.

### 5.2. TracIn’s Influence Results

**Figure 9:**
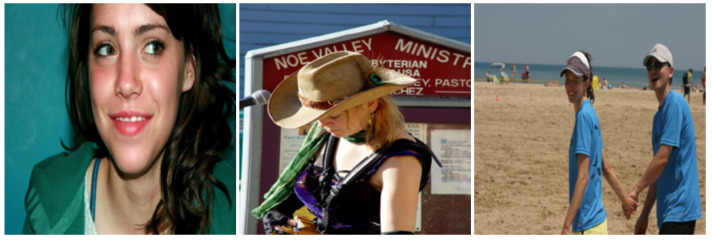
Examples of high-influence training images evaluated using (6) from TracIn, particularly those featuring people or groups that played a key role in distinguishing ASD-related gaze patterns from neurotypical behaviors.

The TracIn influence scores described in Section 4.2 were applied to the dataset to identify the most relevant classes of images that contributed to the network’s learning process. As shown in Figure 9, some of the most recurrent and influential images for the ASD class provided crucial insights into the patterns that are most effective for distinguishing ASD from TD. Specifically, the analysis revealed that images featuring people or groups of people were the most impactful training samples for classifying the ASD label: this aligns well with existing research in clinical psychology and neuroscience, which consistently shows that individuals with ASD exhibit atypical gaze patterns, characterized by reduced attention to socially relevant stimuli, such as faces and people, in comparison to their neurotypical ones. This phenomenon is well-documented in the literature, where gaze fixation patterns are considered a reliable biomarker for early detection of ASD, aiding in the differentiation between ASD and TD behaviors, as described in Section 2.1.

Atypical gaze behaviors, such as fewer fixations on faces or reduced eye contact, are considered key markers for ASD, as they reflect differences in social attention and cognitive processing. To this end, studies have demonstrated that individuals with ASD tend to focus more on peripheral objects or less socially informative regions within a scene, which can provide a window into their distinct cognitive and perceptual processes; these gaze patterns not only aid in identifying social deficits but also offer insights into broader behavioral traits, such as reduced engagement in social interactions and difficulties in interpreting facial expressions. For example, prolonged fixations on non-social objects may indicate a preference for static, less dynamic stimuli, which contrasts with the more fluid and socially driven gaze behaviors typically observed in TD individuals. Also, the use of gaze patterns as a diagnostic tool is supported by numerous studies that leverage eye tracking technology to quantify these behavioral differences as explained in Section 2.1 by analyzing such data, and machine learning models can be trained to distinguish ASD from TD based on these subtle but significant differences in visual attention.

The application of TracIn allowed us to quantify the influence of specific training images, providing a clear validation that the NN is learning meaningful distinctions that align with clinical research. By leveraging TracIn, we identified which training samples most effectively influenced correct classification, highlighting a preference for images featuring human faces or interactions. This further supports the hypothesis that altered social attention is a core feature of ASD. Additionally, the analysis underscores the role of scanpaths related to images of people as the most informative features, corroborating findings from the existing medical literature that emphasize differences in visual attention between individuals with ASD and those who are typically developing. This confluence of ML outcomes with clinical insights demonstrates how advanced data attribution techniques can enhance our understanding of model behavior, providing transparency that is essential for medical AI applications.

## 6. Conclusions

In this paper, we presented the GBAC, an NN model that leverages eye tracking data and the TracIn method to classify ASD and provide insights into the visual attention behaviors associated with the condition. By incorporating TracIn to compute self-influence scores, we were able to filter out noisy or anomalous data, which significantly enhanced both model accuracy and efficiency. The experimental results clearly demonstrated that the self-influence-based reduced dataset GBAC-SIST consistently outperformed models trained on the full dataset GBAC-FT and on randomly reduced datasets GBAC-RST, as well as all the benchmark models. Specifically, the GBAC-SIST model achieved a test accuracy of 94.35% and an F1-score of 0.9315, while using only 77% of the available data, showcasing the effectiveness of selectively removing outliers to improve generalization.

Looking ahead, several promising directions for future research emerge. One avenue involves the development of a portable diagnostic device that integrates the GBAC methodology into a real-time hardware solution. This device could combine eye tracking systems with on-device NN processing, allowing for early and accessible ASD screening in clinical and non-clinical settings. Another important direction is to ensure that the model generalizes across different populations by expanding the dataset to include a more diverse range of subjects across various age groups and geographical regions. Additionally, while TracIn proved effective in our experiments, future work could explore the impact of alternative data attribution methods, such as TRACK [9], on both model performance and explainability. Lastly, integrating additional data modalities, such as gait analysis, fMRI, or EEG, alongside eye tracking data, could provide a more comprehensive diagnostic approach to ASD, potentially improving the robustness and depth of the classification models.

## Figures and Tables

**Table 1:** Main hyperparameters of the GBAC model.

Component	Hyperparameter	Value
	Number of Conv Layers	4
	Filter Sizes	[32, 64, 128, 256]
CNN (Images)	Kernel Sizes	[3, 3, 3, 3]
	Activation Function	ReLU
	Pooling	Avg/Max
	Number of Conv Layers	4
	Filter Sizes	[32, 64, 128, 256]
CNN (Fixation Maps)	Kernel Sizes	[3, 3, 3, 3]
	Activation Function	ReLU
	Pooling	Avg/Max
	Number of Layers	2
	Hidden Size	1024 (both layers)
LSTM	Dropout	0.3 (both layers)
	Input Size Layer 1	2
	Input Size Layer 2	1024
Fully Connected Layers	Number of Layers	4 (1 per branch, 1 final)
	Activation Function	Sigmoid (final)
Optimizer	-	Adam
Loss Function	-	Binary Cross-Entropy Loss

**Table 2:** Performance metrics of the GBAC models, comparing the full dataset model (GBAC-FT) with self-influence-based (GBAC-SIST) and random-reduced (GBAC-RST) training sets.

Method	Dataset Usage	Test Accuracy (%)	Precision	Recall	F1-Score
GBAC-FT	100%	93.64	0.926	0.918	0.9219
**GBAC-SIST 1**	** 77%**	**94.35**	**0.932**	**0.931**	**0.9315**
GBAC-RST 1	77%	93.18	0.940	0.881	0.9094
GBAC-SIST 2	67%	94.13	0.932	0.911	0.9219
GBAC-RST 2	67%	92.81	0.938	0.897	0.9173
GBAC-SIST 3	50%	92.16	0.927	0.845	0.8848
GBAC-RST 3	50%	91.50	0.904	0.904	0.9040

**Table 3:** Benchmark results comparing the GBAC models with existing ASD classification methods on the Saliency4ASD dataset.

Model	Test Accuracy (%)	Precision	Recall	F1-Score
RM3ASD [33]	68.50	-	-	-
STAR-FC [35]	62.13	-	-	-
AttBasedNet [32]	93.00	-	0.930	-
SP-ASDNet [34]	57.90	0.562	0.592	0.5697
**GBAC-SIST 1**	**94.35**	**0.931**	**0.932**	**0.9315**
**GBAC-FT**	**93.64**	**0.926**	**0.918**	**0.9219**

## Data Availability

Code and datasets will be made available upon request.

## Abbreviations

The following abbreviations are used in this manuscript:

ASD	Autism Spectrum Disorder
ADHD	Attention Deficit Hyperactivity Disorder
DL	Deep Learning
NN	Neural Network
CNN	Convolutional Neural Network
LSTM	Long Short-Term Memory
ML	Machine Learning
TDA	Training Data Attribution
SIST	Self-Influence Selected Training
RST	Random-Selected Training
GBAC	Gaze-Based Autism Classifier
DSM-5	Diagnostic and Statistical Manual of Mental Disorders 5
fMRI	Functional Magnetic Resonance Imaging
EEG	Electroencephalography
Tobii T120	Tobii T120 Eye Tracker
GPU	Graphics Processing Unit
ReLU	Rectified Linear Unit
